# Prevalence and Risk Factors of Colonization with* Staphylococcus aureus* in Healthy Pet Cats Kept in the City Households

**DOI:** 10.1155/2016/3070524

**Published:** 2016-09-28

**Authors:** Karolina Bierowiec, Katarzyna Płoneczka-Janeczko, Krzysztof Rypuła

**Affiliations:** Division of Infectious Diseases and Veterinary Administration, Department of Epizootiology with Clinic of Birds and Exotic Animals, Faculty of Veterinary Medicine, Wroclaw University of Environmental and Life Sciences, Wrocław, Poland

## Abstract

*Staphylococcus aureus, *especially methicillin-resistant* S. aureus *(MRSA), is a significant pathogen in both human medicine and veterinary medicine. The importance of pets as reservoirs of human infections is still poorly understood. This article provides detailed information of a cross-sectional study of a* S. aureus* colonization in clinically healthy indoor cats. The study systematically assessed a number of different anatomical locations for the* S. aureus* colonization and the influence of a range of potential risk factors on the value of the final* S. aureus* colonization rate. The incidence rates observed for cats with at least one site positive for* S. aureus* or MRSA were 17.5% and 6.63%, respectively. The following risk factors were identified: one or more owners working in the healthcare industry (human or veterinary); dogs being kept with the cat under investigation; treatment of the cat under investigation with antibiotics or chemotherapeutics during the previous year. In conclusion, this study revealed a higher prevalence of MRSA than what has previously been reported in healthy pets. A combination of anatomical locations from which the samples were collected had a major influence on the final value of the* S. aureus* colonization rate.

## 1. Introduction

While the term “carrier” has several meanings, it can apply to “any individual that sheds infectious agents without demonstrating clinical signs” [1]. Longitudinal studies in humans distinguish between at least three carriage patterns of* Staphylococcus aureus* in healthy individuals: persistent carriage, intermittent carriage, and noncarriage. The criteria used to identify those carriage patterns are inconsistent and vary from study to study [2]. It has been found that 10–30% of healthy people are persistent* S. aureus* nasal carriers and 70–90% are temporary carriers [3, 4]. Little is known about incidence of* S. aureus* in the community of Poland, but in hospital environments the proportion of MRSA isolates was found to be 22.7% (ranging from 3.7 to 63.1% in individual hospitals) [5]. The incidence of* S. aureus* positive nasal samples across nine European countries was found in one study to be 21.6% (ranging from 12.1% in Hungary to 29.4% in Sweden) [6]. The highest MRSA prevalence in this study was reported in Belgium (2.1%). The nasal carriage of* S. aureus* in humans increases the risk of development,* inter alia*, of some skin diseases [7], wound colonization [8], surgical site infections [9], or respiratory tract infections [10].

*S. aureus* also has zoonotic potential. Dogs and cats, the most frequently kept pets, may play a role in household* S. aureus* transmission and recurrent MRSA infections in humans. Therefore, there is the need to assess the likelihood of this potential by studying the carriage of* S. aureus* in domestic animals and identifying risk factors that may be associated with interspecies transmission. The recognition of these risks may facilitate the design of appropriate colonization and infection control as well as prevention strategies in veterinary practices, with public health benefits also possible for the broader community.

There is a lack of longitudinal studies which deal with the carriage patterns of* S. aureus* in animals, but the same three carriage patterns described in humans probably occur in animals. What limits the comparability of these reports in animals is poor harmonization of sampled materials, bacterial cultures, and identification of* S. aureus* strains.

The present study reports on the incidence of* S. aureus* and MRSA colonization in cats living in close contact with their owners. The objective of the study was to systematically test the sensitivity of different anatomical locations and to identify risk factors for* S. aureus* and MRSA colonization in cats by investigating certain characteristics of the animals and their owners.

## 2. Materials and Methods

### 2.1. Study Population and Sampling Procedures

Animals were recruited as part of a cross-sectional study that targeted only clinically healthy cats from the city of Wrocław, Poland. A primary criterion in order to include a cat in the research sample group being examined was a statement from the owner that the pet was bred in Wrocław and had no outdoor access. The health status of each animal under investigation was assessed on the grounds of a diagnostic interview and physical examination. A healthy cat was one that showed no clinical signs of the disease during the physical examination and interview with the owner. The animals were included in the examination group after receiving approval from the owners to take samples of the cats. Additionally each owner was asked to fill out a survey about the cat being examined and about the household. There were no samples collected from humans.

The research project was submitted to the 2nd Local Ethics Committee for Animal Experiments in Wrocław. Due to the noninvasive samples collection procedure, the Ethics Committee qualified the study as research which did not require the approval of the Ethics Committee. Each cat owner consented to take part in this study and filled out the proper documentation.

To assess the variation of colonization throughout the cat and to identify the best locations to sample in order to detect carriage, or their combinations, swabs were taken from each cat that qualified, as follows: from the conjunctival sacs, nares, anus, and skin (groin). The material was collected from animals by a veterinary physician and placed into 2 mL liquid brain-heart infusion (BHI) medium. The pet owners were asked to fill out a brief questionnaire to investigate factors which could potentially correlate with colonization with* S. aureus.*

### 2.2. Survey Instrument and Data Collected

At the time of the cat screening, each participant had completed a survey questionnaire that held epidemiological data for the 12-month period prior to the animal sampling and covered all the household members. The data were collected with regard to several risk factors for the acquisition of* S. aureus* such as hospitalization of any member of the household (surgery, emergency room visits, and inpatient); occupational risk (employment in human health-care practice, physician, nurse, and hospital porter, or veterinary healthcare, physician and veterinary technician); and the diagnosis of* S. aureus* colonization or infection in the past year in any household resident. The nature of human and animal contact was defined as “close” if the cat had continual access to the same rooms and surfaces as the household members. Additionally some person-pet interaction was noted (feeding and grooming, if the participant allowed the pet to lick the hands or face).

The survey also captured data regarding pet-related factors: age, sex, breed, the presence of other pets in the households, and questions concerning the health of the animal (as well as any treatments the pet may have had in the preceding year, the antibiotic class, and the specific antimicrobial agent when available). Dates were provided by the pet owner in the presence of a veterinary physician. Approval for the study was obtained from the Bioethics Committee in Wrocław (Nr 479/215).

### 2.3. Sample Identification

Swabs were placed in tubes containing 2 mL BHI and were incubated at 37°C for 24 hours; then one microlitre of BHI was subcultured on Mannitol Salt Broth and a blood agar plate (Graso Biotech, Poland). The plates were then incubated for 24 hours. The incubation was extended to 48 hours if the result of the culture was negative or uncertain. The isolates were preliminarily identified by way of colony morphology, gram-staining, and detection of enzyme production (coagulase tube test; IBSS Biomed, Poland). All suspected colonies were further identified using molecular methods.

DNA purification was conducted using the manual phenol/chloroform extraction method with initial digestion using lysozyme (Sigma-Aldrich, USA). The isolates were identified as* S. aureus* by a polymerase chain reaction (PCR) using* S. aureus* specific primers of the* nuc* gene which encodes thermonuclease (*nuc*-f: GAAGATCCAACAGTATATAGTGC and* nuc*-r: ATTGACCTGAATCAGCGTTGTCTT) [11]. The isolates were identified as MRSA by detection of the* mec*A or* mec*C gene by PCR [12]. To amplify the short sequence repeated region of the* spa* gene, specific primers and thermal cycling conditions were used according to Harmsen et al. [13]. The completed reaction mixtures were sent to sequencing services (MacroGene, Netherlands), and the sequences were analyzed using the Ridom SpaServer (http://spa.ridom.de/).

### 2.4. Antibiotic Resistance

All isolates of* S. aureus* were screened for oxacillin susceptibility using *E*-test (MIC Test Strip, Liofilchem, Italy) to determine minimum inhibitory concentration (MIC) to assess the methicillin-resistance of the isolates at the phenotypic level. The results were interpreted according to the manufacturer's instructions. The* S. aureus* isolates were interpreted as MRSA when the* mec*A gene was present, regardless of the MIC concentration according to the CLSI recommendation [14]. All the* S. aureus* isolates in which the presence of* mec*A or* mec*C was not confirmed and which showed resistance to oxacillin using the *E*-test were analyzed for susceptibility to amoxicillin using clavulanic acid (20/10 *μ*g/disc) (Mast Diagnostics, UK) and the disc-diffusion method and were interpreted according to the Clinical and Laboratory Standards Institute (CLSI), document M100-S24 [14]. All suspected borderline oxacillin-resistant* S. aureus* (BORSA) were screened for hyperproduction of beta-lactamase using the Cefinase test (BioMérieux Inc.).

### 2.5. Statistical Methods

To calculate the prevalence and confidence intervals of* S. aureus* and MRSA, the two-step bootstrap method was used. In the first step, 224 replacement households (each with equal probability) were drawn from the pool of 224 households. In the second step, one cat was drawn from each household. This process was repeated 10,000 times. Use of this method enabled the elimination of bias, which could be the result of cats infecting each other in the same household.

The characteristics of the cats and questionnaire answers were compared to the* S. aureus* colonization scores. The data was analyzed using the Wilcoxon test and 2 × 2 contingency tables. *p* < 0.05 was considered to indicate a statistically significant association. Statistical analysis was carried out using the R Statistical Package (*v*. 2.11.1).

## 3. Results

A total of 415 cats were swabbed for colonization from January 2013 to November 2014 at the Department of Epizootiology and Clinic of Birds and Exotic Animals, Faculty of Veterinary Medicine, Wrocław University of Environmental and Life Sciences, Poland, at local veterinary clinics and catteries either during annual check-ups or in the course of preventive vaccinations. Cats were assigned to three groups on the grounds of data obtained from surveys of 224 households: single feline (only one purebred or mixed bred cat in the household); multiple feline (more than one purebred or mixed bred cat in the group, but not in registered cattery); and cat breed (purebred cats in a registered cattery kept in the same condition as pet cats (in city households)). In some cases, more than one cat was swabbed from the household. Detailed data from the animals under investigation is presented in Table 1.

**Figure 1:**
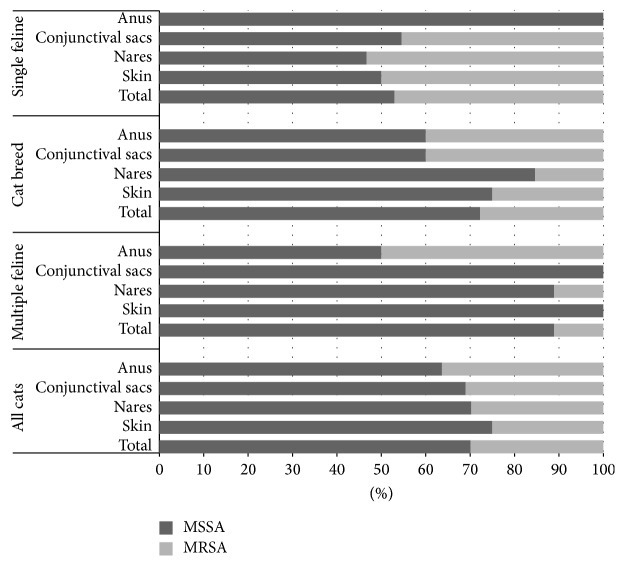
This shows proportion of methicillin-sensitive* Staphylococcus aureus* (MSSA) and methicillin-resistant* Staphylococcus aureus* (MRSA) strains isolated from each body site in different groups of cats according to molecular method.

Animals were considered positive regardless of whether* S. aureus* was isolated from the skin, conjunctival sacs, anus, or nares. The average prevalence of* S. aureus* among all cats under investigation using the bootstrap method was 17.5% (CI: 12.56–22.87%). The prevalence for each group of cats, that is, single cats, multiple cats, and breed cats, was 18.92% (CI: 11.71–27.03%), 16.64% (CI: 9.57–24.47%), and 13.85% (CI: 0–33.33%), respectively. The presence of the* mec*C gene was not confirmed in any of the investigated isolates. The prevalence of MRSA isolates was 6.63% (CI: 3.59–10.31%) and for each group as follows: single cats: 9% (CI: 4.51–14.41%), breed cats: 5.84% (CI: 0–16.67%), and multiple cats: 3.9% (CI: 1.06–8.51%). No significant differences between the groups were detected. The prevalence of MRSA according to MIC results only was lower: only 2.29% (CI: 0.004–0.045%). One BORSA strain was detected in material from the nares. The proportion of methicillin-sensitive* Staphylococcus aureus* (MSSA) and MRSA strains isolated from each body site in different groups of cats is presented in Figure 1.

**Figure 2:**
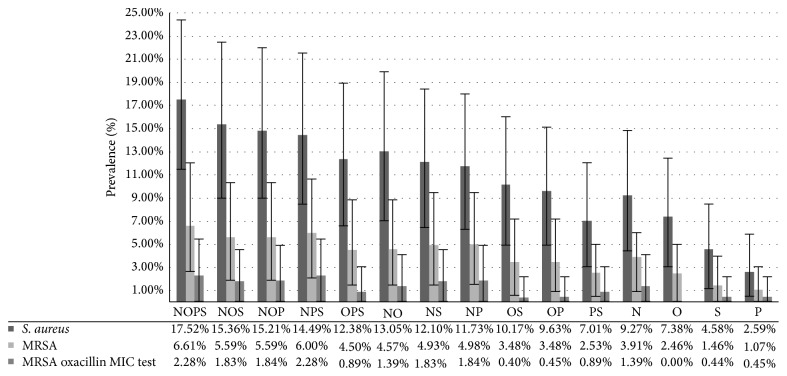
Detailed data of the* S. aureus* and MRSA prevalence according to material sampled in different anatomical locations and their combinations. Sampling places: nares (N); conjunctival sacs (O); anus (P); skin (S). For each combination of sampling places, confidence interval was marked which was calculated using bootstrap method.

The nares were the site most commonly colonized, both for total* S. aureus* isolates as well as for MRSA strains only. Among the cats colonized by* S. aureus* (17.52%), more than the half (9.27%) had the bacterium in their nares. On the other hand, in material from the anus,the lowest number of* S. aureus* isolates was detected. Detailed data about the importance of the residual anatomical location and their combinations are presented in Figure 2.

Two or more* S. aureus* strains were obtained from 20 cats. The detailed data is presented in Table 2. Other isolates were found in the material from only one anatomical location: skin, nares, conjunctival sacs, or anuses. The highest number of* S. aureus* strains was isolated from the nares (*n* = 20) with a similar percentage (around 28%) in each investigated group. In twenty-nine cats,* S. aureus* was isolated from only one sampling site such as the skin (9 cats), conjunctival sacs (13 cats), and anus (7 cats).

Isolates were characterized into 39* spa* types. Among all isolated* S. aureus* strains the most frequently observed spa types were t091 (26%), t008 (8%), and t002 (6%). All of them were previously reported in human in Poland (http://www.spatialepidemiology.net/srl-maps/ and http://spa.ridom.de/frequencies.shtml). No correlations were observed between* spa* types and the anatomical location of the* S. aureus* isolation or the affiliation of the cat to any of the investigated groups. Twelve cats were colonized in two or three anatomical locations with the same* spa* type (Table 2).

Surveys concerning the cats and household were completed by each cat owner. All the owners declared that the cat under investigation had close daily contact with household members. There were on average three residents in a typically investigated household. Cats were colonized with* S. aureus* in households where the number of residents was lower (fewer than 3). In 20.54% households, one or more residents of the household were hospitalized, but this did not have an influence on the study results. In the households with occupants working in human or veterinary healthcare, cats were at a higher risk of colonization by* S. aureus* and MRSA. When more than one pet was kept in the same household, the animals were in contact with each other or they had access to the same facilities. Only the presence of dogs in the household increased the risk of* S. aureus* or MRSA colonization in cats. The last risk of colonization was connected with the previous treatment of an investigated animal; however, such a correlation was not observed when other pets in the household were treated. The most important statistical analysis results of risk factors are presented in Table 3.

## 4. Discussion

While a number of studies have examined the prevalence of staphylococci among companion animals, there is a lack of studies focused on the colonization of only healthy pets (without clinical signs) who have daily close contact with their owners. A comparison of* S. aureus* carriage rates reported by different studies is problematic because of the various sampling strategies and isolation methods used for assessing staphylococcal carriage. Our results were lower than those obtained by Abraham et al. [15] where the colonization of* S. aureus* was confirmed in 34% of clinically healthy cats and 58% of cats with inflammatory skin diseases (ISD). In this study, MRSA strain isolates were reported in 7% of healthy cats and 20% of ISD cats, respectively. Similarly, a high prevalence among cats was noted by Lin et al., 39.6% [16]. Thus, a high prevalence in those studies could be caused by too small sample size and a larger number of anatomical sampling places (six places were used by Abraham et al. [15] compared with our research). The larger number of anatomical sampling places increases the probability of detection bacterial strain and may influence the final value of prevalence. In contrast, the results obtained by Iverson et al. [17] are very similar (15%) to ours, although the authors did not detail precisely the health status of the sampled pets. The prevalence of MRSA in mentioned study was 8% which is similar to Morris et al.'s results [18] where the prevalence of MRSA in pets was 11%. These results could have been influenced by household MRSA exposure. The average MRSA prevalence obtained in our research was 6.63%; however, we did not check colonization of the owners who could be the possible source of MRSA for cats. The highest MRSA prevalence was obtained in the single feline group, although there were no statistically significant differences between the cat groups. Usually in similar reports, the prevalence of* S. aureus* among cats and dogs is about 10% or lower and MRSA prevalence does not exceed 4% [4, 16, 19–21]. However, the MRSA prevalence according to the oxacillin MIC only was very similar to previous studies worldwide in which phenotypic method was used. Nevertheless, prevalence based on the* mec*A gene presence shows that the molecular method is much more sensitive and should be included in routine diagnostics.

The majority of the reports about the prevalence of* S. aureus* are based only on one sample place. There is an assumption that nasal or anterior nares swabs obtain the most appropriate material for* S. aureus* carriage investigation. Many studies on* S. aureus* carriage in humans use a cross-sectional design with a single nasal culture to classify an individual as a carrier or not [3, 4, 22, 23] although multiple body sites can be colonized in human beings [2]. Predominant sites of colonization in cats are not known. In dogs,* S. aureus* isolates were obtained from nares, eye, ear, reproductive extremity, urine, abscess, skin, and throat [24]. Nevertheless, anterior nares swabs are most frequently taken to obtain material from pets under investigation [25–27], although, as described, the increase in the number of anatomical locations which are sampled also increases the chance for* S. aureus* isolation.

In some reports, more than one anatomical site was used to collect samples but recommendations of body site choices are not available. Usually, they are the combinations of nasal and rectal, perineal, or mouth swabs as described, with additional sites also recommended [4, 15, 17, 28–30]. Moreover, variable results can be obtained with some animals that are positive at only one sample site [31]. Our study shows that the combination of anatomical places of sampling has a major influence on the final results. In our research we used new anatomical locations which had not previously been investigated in this type of study. The nares and the conjunctival sacs, respectively, were the two most common colonization sites. Several reports show that staphylococci are frequently isolated from the conjunctiva of clinically healthy cats [32]. Additionally, importance of the conjunctival sac samples is only about 2% lower compared to the nares as a single place of sampling.

This study provides a comprehensive investigation of risk factors for* S. aureus* and the MRSA colonization of cats. The inclusion criterion of this study that only cats without outdoor access were examined decreased the bias of the assumption that the colonization of the cat could be caused by close contact with household members, contact with other animals kept in the same environment, visiting veterinary clinics, or treatment with chemotherapeutics or antibiotics. During the survey, owners were asked about these issues. We identified the following risk factors: the previous (one-year) identification of* S. aureus* in any individual in the household (owner, cat, and other animals); one or more owners working in healthcare or in veterinary healthcare; dogs being kept with the cat under investigation; the treatment of the cat under investigation with chemotherapeutics in the past year. We have not confirmed that the hospitalization of an owner in the last year had an impact on the* S. aureus* colonization in cats. Nevertheless, inpatients are at a higher risk of harboring MRSA, and the transmission of MRSA between companion animals and colonized humans has been widely described in other reports [24, 33, 34]. Our results could be influenced by the relative sparseness of the group of owners who were inpatients during a set period of time (only about 20% of households).

The influence of the household members' occupations has been discussed in several research papers. In particular health-care workers' pets are at higher risk to be carriers of* S. aureus* strains [24] though in Kottler et al.'s report [4] no differences in the prevalence of MSSA or MRSA between healthcare workers and nonhealthcare workers' households were found. Nevertheless, it has been previously confirmed that veterinary and healthcare staff are at higher risk of being carriers of MRSA strains [4, 29] and, taking into account the possibility of horizontal transmission of the pathogens between human host and pet, this risk is very probable. One statistically significant factor was treatment during the past year. It has also previously been described that the number of antimicrobial courses is a risk for* S. aureus* infection and the carriage of multidrug resistance strains in dogs and cats [30, 35]. Additionally, we also found that cats that are kept in the same household as dogs are in a risk group. This type of correlation was not found with the number of cats or other animals bred in these households. As this risk factor has not previously been reported, it should be further investigated. Similar to other reports, there was no correlation between characteristics such as age, breed, and sex in relation to* S. aureus* colonization [35]. There is a need to add that all risk factors were reported by owners which could introduce a bias into the study. We tried to avoid this situation by assisting the owners while completing the survey.

## 5. Conclusion

Our study has shown that the colonization of healthy cats with* S. aureus* is common. The sampling design used in this investigation facilitated material from a large number of households, although in further studies all members of the household (people and animals) should be sampled to provide a comprehensive and accurate analysis of colonized households and to check risk factors. Future research should elaborate on the fact that sampling in only one anatomical location may lead to false negative results. It is especially important when the aim of the sampling is the identification of the animal, which may be a source of recurrent infection in owners. Further studies should address the duration of colonization in pet animals, the influence of anatomical sampling places, and protocols for the prevention of* S. aureus*, especially MRSA transmission among species.

## Figures and Tables

**Table 1:** Characteristics of the investigated population.

Category	Number of investigated households	Number of cats	Size of group per household		Sex		Breed		Age								
			$\overset{-}{x}$	*σ*	Female (%)	Male (%)	Crossbreed (%)	Pure breed (%)	≤6 (month)			7–36 (month)			≥37 (month)		
									%	$\overset{-}{x}$	*σ*	%	$\overset{-}{x}$	*σ*	%	$\overset{-}{x}$	*σ*
Multiple feline	94	121	2.7	1.5	51.2	48.8	66.1	32.9	9.9	4.7	1.6	40.5	16.8	9.6	49.6	83.4	39.2
Cat breed	18	182	11.8	6.4	61.0	39.0	—	100	24.2	2.9	1.1	46.7	22.4	9.6	29.1	70.9	27.8
Single feline	112	112	—	—	53.6	46.43	66.1	33.9	10.7	5.1	1.3	60.7	17.4	9.3	28.6	96.5	42.6

$\overset{-}{x}$: arithmetic mean.

*σ*: standard deviation.

**Table 2:** *Spa* type, antimicrobial-resistance pattern of *S. aureus* isolates in cats from which more than one *S. aureus* strain was isolated.

Category	Nr	Characteristics of the cat			Anatomical location of sampling											
					Skin			Nares			Conjunctival sacs			Anus		
		Breed	Age (mth)	Sex	*S. aureus*	Spa type	Oxacillin MIC	*S. aureus*	Spa type	Oxacillin MIC	*S. aureus*	Spa type	Oxacillin MIC	*S. aureus*	Spa type	Oxacillin MIC
Multiple feline	1	Crossbreed	10	M	MSSA	t008	0.25	MSSA	t008	0.125	MSSA	t8420	0.125			
	2	Crossbreed	3	M	MRSA	t002	>256	MRSA	t002	0.19	MRSA	t854	0.125			
					MSSA	t159	0.094									
	3	Crossbreed	48	M	MSSA	t002	0.125				MRSA	t10367	0.125	MSSA	t304	0.38
	4	Crossbreed	54	F	MSSA	t13008	0.094				MSSA	t1171	0.094			
	5	Crossbreed	72	F	MSSA	t775	0.047				MSSA	t9428	0.25			
	6	Exotic	156	M							MSSA	t13654	0.25	MSSA	t223	0.094

Cat breed	1	Persian	84	F	MSSA	t4474	0.047	MSSA	t4474	0.064	MRSA	t4474	0.064	MRSA	t091	0.19
	2	Persian	4	F	MSSA	t091	0.094	MSSA	t091	0.094	MSSA	t091	0.047			
	3	Persian	3	M	MSSA	t091	0.38	MSSA	t091	0.19	MSSA	t091	0.19			
	4	Devon Rex	3	M	MSSA	t091	0.75	MSSA	t091	0.19				MSSA	t091	0.19
	5	Persian	24	F				MRSA	t4474	0.75	MSSA	t4474	0.064			
	6	Persian	84	F				MSSA	t7568	0.19	MSSA	t091	0.125			
	7	Devon Rex	3	M				MSSA	t091	0.38				MSSA	t091	0.047

Single feline	1	Crossbreed	36	F	MRSA	t8420	0.125	MSSA	t8420	0.047	MSSA	t8420	0.094			
	2	Maine Coon	72	F	MSSA	t425	0.19	BORSA	t298	6						
	3	Crossbreed	24	F				MSSA	t008	0.25	MSSA	t008	0.19			
	4	Crossbreed	12	F				MRSA	t002	0.25	MRSA	t002	0.125			
	5	Crossbreed	8	M				MRSA	t008	0.19	MRSA	t005	0.38			
								MSSA	t005	0.25						
	6	Crossbreed	5	M				MRSA	t521	0.094	MRSA	t7482	0.19			
	7	Crossbreed	12	M				MSSA	t11455	0.25	MSSA	t700	0.25			

M: male; F: female.

MSSA: methicillin-sensitive *Staphylococcus aureus*; MRSA: methicillin-resistant *Staphylococcus aureus*; and BORSA: borderline oxacillin-resistant *Staphylococcus aureus*.

**Table 3:** The statistical analysis results of risk factors associated with the colonization of *S. aureus* in cats under investigation.

Variable	Test	*S. aureus*			MRSA		
		*p* value	OR	95% CI	*p* value	OR	95% CI
Breed (crossbreed or pedigree)	Chi-squared^*∗*^	0.29	0.728	0.43–1.24	0.21	0.537	0.23–1.27
Age	Wilcoxon	0.26			0.12		
Sex	Chi-squared^*∗*^	0.46	1.26	0.74–2.13	0.53	1.43	0.61–3.4
Number of the household residents who had close contact with the cat under investigation	Wilcoxon	**0.022** ^*∗*^			0.08		
The family member works in healthcare or in veterinary healthcare	Chi-squared^*∗*^	**0.0032** ^*∗*^	2.29	1.33–3.92	**0.019** ^*∗*^	2.9	1.23–6.95
Hospitalization of an owner in the previous year	Chi-squared^*∗*^	0.075	0.457	0.18–0.98	0.12	0.212	0.01–1.03
Diagnosis of *S. aureus* colonization in the previous year: in household resident or cat under investigation or other animals kept in the household (confirmation of the colonization using laboratory methods)	Chi-squared^*∗*^	**0.013** ^*∗*^	1.56	0.92–2.63	**0.016** ^*∗*^	3.49	1.26–8.76
Number of animals kept in the same household							
Dogs	Wilcoxon	**0.0078** ^*∗*^			**0.017** ^*∗*^		
Cats	Wilcoxon	0.24			0.16		
Others	Wilcoxon	0.76			0.87		
Treatment of cat under investigation in the previous year	Chi-squared^*∗*^	**0.00056** ^*∗*^	2.58	1.51–4.4	**0.015** ^*∗*^	3.01	1.28–7.31
Treatment of other pets in the previous year	Chi-squared^*∗*^	0.65	1.17	0.69–1.97	0.75	1.26	0.53–2.99

MRSA: methicillin-resistant *Staphylococcus aureus*; *p* value: probability value; ^*∗*^results statistically significant; Chi-squared^*∗*^: degrees of freedom is 1.
